# Media and information literacy as a model of societal balance: A grounded meta-synthesis

**DOI:** 10.1016/j.heliyon.2024.e25380

**Published:** 2024-02-01

**Authors:** Hasan M.H. Mansoor

**Affiliations:** Mass Communication Department, College of Humanities and Social Sciences, King Saud University, Riyadh, Saudi Arabia

**Keywords:** Media and information literacy, Lifelong learning, Media in education, Social media, 21st century abilities

## Abstract

Concerns about the spread of disinformation, information disorder, and fake news have grown to unprecedented proportions in recent years. This study aimed to explore how to mitigate this communication disorder and achieve a balance in the relationship among the public, the media, the dominant institutions, and the digital influencers in society. This study used the grounded meta-synthesis method, which relies on induction, to arrive at a new model according to the objective of the study. The process of open, axial, and selective coding included 101 studies, books, reports, and guides, starting with the *Public Opinion* by Walter Lippmann, issued in 1922, and ending with the 2022 Edelman Trust Barometer. The results led to the proposal of a new model to reduce communication dysfunction, in which media and information literacy (MIL) plays a crucial role in increasing an individual's ability to resist disinformation and enhancing their ability to monitor the performance of institutions, as well as expanding the circle of influencers in social media. To fulfil the three goals and contribute to achieving a degree of functional balance in communication within societies, the model recommends enhancing MIL. Other intervening variables, such as the fragility of political, cultural, and legal structures, should not be disregarded.

## 1 Introduction

In his seminal work “*Public Opinion*”, published over a century ago, Walter Lippmann delineated three facets of societal imbalances prevalent within the American context:

⁃ Public opinion is susceptible to manipulation, rendering it unqualified for participation in governance. Lippmann argued that the majority of the public lives in pseudo-environments constructed from stereotypes and distorted symbols, which are exploited through propaganda and deception.
⁃ The press (the only mass media at that time) was criticized for not presenting the truth, and a distinction was made between “news” and “truth”. It was believed that the press was influenced by advertisers and dominant powers.
⁃ Leaders, governments, and political parties manipulate the public through the manufacture of consent by using heroes and celebrities as simplified symbols for interpreting the complex world [1].

In the field of communication, while Lippmann suggested that addressing this problem could be achieved through education and developing citizens' critical abilities in dealing with the press, he considered it a difficult and elusive process due to the inadequacy of the educational system. He argued that “our knowledge of human institutions is still extraordinarily meager and impressionistic. The gathering of social knowledge is, on the whole, still haphazard …” [1] (p.408).

The following questions have arisen more than a hundred years after Walter Lippmann's book:

1. Is public opinion still unqualified to participate in decision making because it lacks information and the ability to analyze and evaluate like experts?
2. Do media outlets still hold a dominant position in presenting a false reality, manufacturing distorted symbols, and deceiving and misleading the public?
3. Does human knowledge in the social and political sciences still stand at the point where, according to Lippmann, we are unable to rely on education as a primary remedy to the dysfunction of the media and the political and economic institutions’ roles in society?

From Lippmann's book (1922) [1] to the Edelman Barometer (2022) [2], it appears that the media's dysfunction continues to be a problem plaguing the world.

The Edelman Trust Barometer is an extensive study involving 36,000 participants from 28 countries, and it concluded that trust levels in governments and the media have diminished, and concerns about misinformation have increased unprecedentedly compared to previous years.

Dysfunction is “the fact of a part of the body not working normally, the situation when the relationships within a society, family, etc. are not working normally” [3] (p.458).

Communication dysfunction takes two main forms:

First, it occurs when media outlets fail to fulfill their expected roles and functions in serving society.

Second, it occurs when media outlets become harmful and have negative effects on society [4].

Dysfunction is countered by the efficiency and effectiveness of performing communication functions. It is also countered by functional balance, which refers to the non-monopolization of influence and the mutual reliance between social units in fulfilling their expected functions. Media outlets are assumed to be social units that perform a set of repeated activities to fulfill societal needs. The relationship between the media and other systems and units in society should be based on mutual reliance to ensure social stability and equilibrium. However, in many cases, conflicts and dominance by certain elite groups and influencers occur at the expense of the public. The tidal wave of irresponsible information is “a threat to social cohesion both within and between countries” [5].

In modern times, communication dysfunction has also been expressed by the term “information disorder”, which is spreading in various forms. The common denominator among these forms is that they do not serve the public good or societal stability, whether it is misinformation, disinformation, or malinformation [6]. Information disorder occurs to varying degrees in these three cases, as the proliferation of such information disrupts the genuine functions of the media and exacerbates dysfunctional aspects.

The term “fake news” has become widely used in media, political and academic discourse. It is an inaccurate term in describing the intricacies of information disorder. It is vital to discern between different sorts of this information based on their authenticity or incorrectness as well as whether they are published by accident or with the aim to harm. Treatment and preventative methods will differ as a result.

“Fake news has become a buzzword especially after the 2016 presidential elections in the United States” [7] (p.147) It has been commonly used in the context of political leaders' attacks against media outlets (for example, Donald Trump). The term has been used to describe a number of different phenomena: news satire, news parody, fabrication, manipulation, advertising and propaganda [7]. Fake news, mis- and disinformation are not a problem of a particular country but are found in politics around the world [8].

This study agrees with Bounegru and her colleagues [9] and Wardle & Derakhshan [6] that the term (information disorder) is more appropriate and complete in characterising the prevalent communication problem than the term (fake news) However, since the extensive use of these phrases, this study will also employ the terms (fake news) and (misinformation) as keywords to access relevant studies and literature.

This study aims to bridge the knowledge gap in this field by extrapolating and tracking scientific approaches that addressed the phenomenon of information disorder and communication dysfunction in order to answer the following main question:

How can the dysfunctionality in traditional and digital media be reduced and a certain balance be achieved in the relationship between the public, the media, the dominant institutions, and the influencers in society?

In qualitative studies that adopt the grounded theory approach, the research question begins as a general and broad question, and then it becomes more specific and focused during the research process as the concepts and relationships become clear. Therefore, we started with a general and open question, but it was not open to the extent that it allowed all possibilities, nor was it narrow or focused to the extent that it prevented the discovery of the phenomenon. Qualitative research—as in this study—does not target testing hypotheses, but the question in qualitative research is “a statement that identifies the phenomenon to be studied. It tells the readers what the researcher specifically wants to know about this subject” [10] (p.41). Moreover, a systematic data analysis can provide opportunities to build a relevant (decisive) theory to answer the main research question [11].

## 2 Methodology

This study was based on the grounded theory approach, which is a general methodology for building theories. It represents one of the qualitative research methods that use systematic procedures to develop and build a theory in an inductive manner about a particular phenomenon [12].

The primary aim of grounded theory methodology is not to explain or predict behavior as scientific theories do. Rather, it is a methodology that provides us with “reasoned normative models—rational reconstructions—to inform praxis and critique” [13] (p.265).

It is a qualitative research method that uses a set of procedures to develop “an inductively derived theory about some phenomenon” [14] (p.229).

Studies employing grounded theory methodology have utilized qualitative research tools such as in-depth interviews, observations, and focus groups [[15], [16], [17]], as well as media content analyses [18,19]. Other studies have adopted grounded meta-analysis methodology [[20], [21], [22]].

The methodological approach of a meta-analysis is contrasted with another approach, called a meta-synthesis. The former is an inferential method with the ultimate goal of verifying and refining theory and shedding light on knowledge gaps. It begins with specific and repeatable research terms and treats previous studies as a research community. The criteria for selecting previous literature include the ability to account for effect size alongside specific criteria for the review, which should include the research quality. The data include study characteristics and quantitative findings—explicit and clear data. On the other hand, a qualitative meta-synthesis is an inductive approach with the ultimate goal of theory-building and identifying knowledge gaps. Based on the research questions, samples are determined randomly, purposely, or through a study community, and the criteria for selecting previous literature are determined based on the research questions, the underlying theory behind the work, and clear methods. The data include qualitative findings, the study context, the methods, the analysis, the researcher's position, the conceptual framework, and the data sources.

In a meta-synthesis, the “analysis can consist of key findings within a context discussed through ethnography, grounded theory, case study, phenomenology, or discourse analysis … Results consist of new theory, encompass common as well as abnormal phenomena, remain unfinished until applied in a new setting” [23] (p.526).

There is often confusion between literature reviews and a meta-synthesis. “Literature reviews situate a reader in the context of what has been previously studied, while meta-synthesis uses sophisticated methods of inductive interpretation to report summative findings or new theory” [23] (p.530).

The current study adopts grounded meta-synthesis methodology to develop a new model according to the study's goal. It embraces a qualitative analysis rather than a quantitative analysis of the previous literature.

Grounded theory methodology provides an appropriate framework for meta-synthesis studies. However, it has been underutilized until recently, with a few studies adopting this approach, such as those of Benoot and Hannes (2014) and Rees et al. (2022) [24,25].

The current study benefits from the critical realism philosophy in its methodological application along with grounded theory, as demonstrated by Hoddy (2019) [26]. Critical realism is a philosophy in the social sciences founded by the British philosopher Roy Bhaskar (1944–2014). It has become one of the significant philosophical frameworks underlying social scientific research, aiming to uncover the underlying mechanisms behind social problems and explain and elucidate phenomena that impact human reality. It fosters the conviction of the need to change reality by addressing the mechanisms that cause these phenomena and problems.

The critical realist grounded theory, as a new approach, combines the philosophical framework of critical realism with the methodological approach of grounded theory. “Critical realism and grounded theory then become highly compatible, sharing a focus on abduction and commitment to fallibilism and the interconnectedness of practice and theory” [27] (p.371).

The research project, according to the critical realist philosophy, consists of four stages that correspond to four methodological techniques of grounded theory. These stages are as follows:

1. Description: This stage involves describing the event or situation based on people's interpretations and reasons.
2. Analytical resolution: This stage focuses on identifying the fundamental elements/components of the phenomenon.
3. Abduction and retroduction: In this stage, the components are re-described in terms of theories related to relationships and structures, proposing mechanisms that may influence them.
4. Concretization and contextualization: This stage examines the shared impact of abstract components in concrete situations.

These four stages are accompanied by four methodological techniques in grounded theory:

- A pre-data-collection literature review;
- Theoretical sampling, open coding, and a comparative method;
- Theoretical sampling, initial axial coding (diagramming), and a comparative method;
- Axial coding alongside a continuous literature review and comparisons [26] (p.115).

It is worth noting that the fourth stage of the critical realism project is concerned with testing the theory after it has been constructed in the first three stages. Therefore, the grounded theory primarily provides methodological mechanisms for the first three stages, while the fourth stage in critical realism, which is the testing of the theory, requires a complementary research effort.

### 2.1 Data collection

According to grounded theory methodology and the meta-synthesis approach, there is no specific limit for determining the sample size of previous studies and literature. It is considered sufficient when the researcher reaches a saturation level, as “search criteria and what studies to include in a qualitative meta-synthesis are determined by the focus of the research questions” [23] (p.532).

The current study relied on searching the relevant literature to identify and construct concepts that formed categories. By understanding the relationships between the main categories, the model was developed, as detailed in the coding procedures. The selection of the literature and previous studies included in the coding process comprised various sources, such as books, articles, reports, and guidelines, focusing on media functions, dysfunction, and the phenomenon of information disorder.

Keyword-based research was conducted in scientific databases. No specific time frame was set, as the topic of communication function and dysfunction dates back to the early writings of communication research, politics, and sociology pioneers, such as Walter Lippmann (1922) [1], and continues to this day.

The keywords used aligned with the main research question and included “media functions, communication dysfunction, misinformation and fake news, and information disorder”.

The literature search was conducted on December 12–13, 2022, using five databases: Web of Science, EBSCO, AlMandumah, ProQuest Ebook Central, and Google Scholar.

From each database, the first 20 results for each keyword were collected, resulting in a total of 400 results across the five databases. After removing duplicate records, the final number of relevant references was 213. These references were examined by reading the abstracts, and 112 were excluded due to them not being directly related to the main objective of the current study. Thus, the analysis included 101 previous studies, books, reports, and guidelines, which were arranged in chronological order from Walter Lippmann's book “*Public Opinion*, 1922” to the Edelman Trust Barometer, 2022 [1,2].

**Fig. 1:**
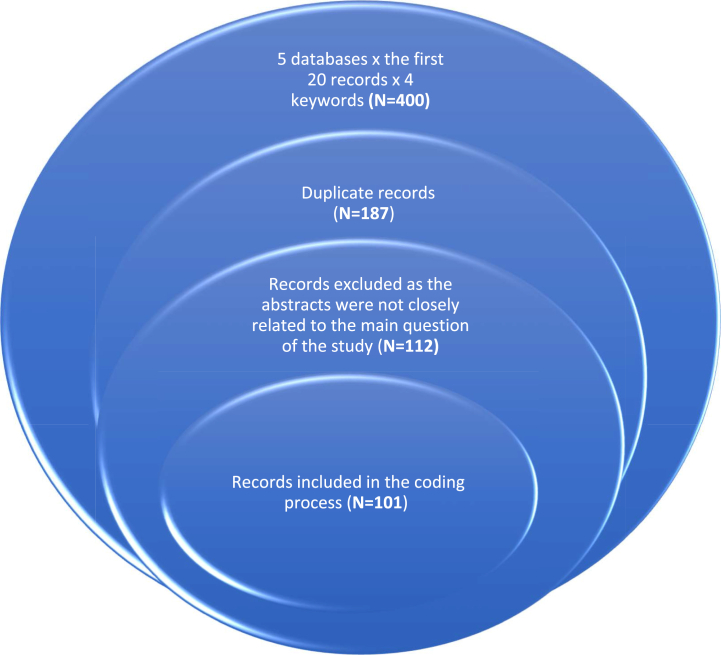
The literature inclusion procedures.

Fig. 1 shows the literature inclusion procedures, from the identification stage in the two wider circles to the screening stage (the third circle), and ending with the included stage, which is the narrowest circle.

### 2.2 Sample characteristics

Although the method of organizing the search results from databases relied on the option of reviewing and sorting the results based on their relevance to the keywords rather than their publication dates (sort by relevance), it is noteworthy in the distribution of literature that, despite covering a hundred-year period (1922–2022), over 74 % of the references were published in the last 25 years, as indicated in Table 1.

**Table 1:** Chronological distribution of the study sample.

Publication date	Article/Book/Report/Guide	
	n	%
1922–1946	1	0.99
1947–1971	9	8.91
1972–1996	16	15.84
1997–2022	75	74.26
Total	101	100

This logical result indicates the increasing interest in the phenomenon of information disorder and the spread of fake news in recent years after the emergence of the internet and social media.

### 2.3 Data coding design

According to the grounded theory methodology, data analyses and coding are conducted in three main stages. In the first stage (open coding), the researcher identifies concepts and their characteristics and dimensions. In the second stage (axial coding), the main and sub-categories are linked. The third stage (selective coding) involves explaining and interpreting the story (theory) as a whole [[10], [11], [12],^28^].

Open coding is an analytical process that involves identifying concepts and discovering their characteristics and dimensions within the data. It also involves identifying various categories, with some related to phenomena, while others (later becoming sub-categories) refer to situations, actions, interactions, or outcomes. The conceptual labels placed on the categories do not necessarily indicate whether the category refers to a situation, action and interaction, or outcome. Each category or sub-category has a distinct set of clear characteristics and dimensions.

In the subsequent analytical steps, the data are reassembled through phrases that describe the nature of the relationships between different categories and their sub-categories. These relationship phrases are commonly referred to as “hypotheses”. The resulting theoretical framework allows us to generate new interpretations about the phenomena [10] (pp.101–103,129).

In the axial coding stage, the main categories are linked to their sub-categories. This stage is called “axial” because the coding revolves around the axis of a category, connecting categories at the level of properties and dimensions. “A major purpose of axial coding is to bring the data back together again into a coherent whole after the researcher has fractured them through line-by-line coding” [29] (p.603).

In this study, the main ideas included in the previous literature (Table 2) were sorted and arranged into five parts that fit the research topic as Table 3 shows in the open coding stage: The expected functions of communication in society, dysfunctions, opportunities, challenges, and stakeholders as individuals or institutions.

**Table 2:** The main ideas of the previous literature.

	Previous Literature	The main ideas
1	Lippmann, 1922 [1]	Most of the public lives in a pseudo-environment, based on stereotypes and distorted symbols, and is exploited by propaganda and deception. The press does not present the truth. Leaders, governments, and parties manipulate the public through the manufacture of consent and the creation of stars. Access to and awareness of reality must be limited and restricted before propaganda creates a fake environment. Education is the supreme remedy by developing the citizens' ability to deal with the press critically, but it is a far-reaching process. Education is the optimal approach to tackling fake news as a “vaccination” offering resistance against the worst cases of fake news and post-truth. Dealing with fake news and disinformation would dial down the temperature of political and social debates – and confrontations – would improve trust in societies and would contribute to a more healthy environment.
2	Lessenski, 2021 [30]	
3	Lasswell, 1948 [4]	The functions of communication involve surveillance of the environment., community integration, and transmission of social heritage across generations. Media become dysfunctional or harmful to society when they are used for misleading purposes. Dysfunction occurs when communication fails to fulfill its expected roles and responsibilities in serving the community or when it has a negative impact on society.
4	Lerner, 1958 [36]	Communication functions in modernizing societies, and empathy. Communication has increased the aspirations of individuals; Unachievable aspirations has become a revolution of rising frustrations
5	Lerner 1963 [37]	
6	Lazarsfeld & Merton, 1960 [38]	Communication functions in facilitating the exchange of views in the community to confirm the legitimacy of the status, maintaining and strengthening social standards. The narcotizing of mass media as a dysfunction.
7	Wright, 1960 [39]	Communication functions: entertainment, mobilization, political and commercial advertising, information, analysis and interpretation, education and socialization, persuasion and public relations
8	Mendelsohn, 1966 [40]	
9	Hiebert et al., 1979 [41]	
10	Rogers, 1962 [42]	Diffusion of innovations. The transition of societies from the traditional stage to the modernization stage
11	Schramm, 1964 [43]	Surveillance of the environment. Political function. Socialization.
12	Tichenor, Donohue, & Olien, 1970 [44]	The flow of communication messages from the media leads to audience segments with higher economic and social levels acquiring information at faster levels than segments with lower levels, Knowledge Gap between these social segments increases.
13	Weaver, 1972 [45]	Television enhances the odds of detachment (at best) or cynical rejection (at worst) towards the political institutions in society.
14	Robinson, 1976 [46]	TV news is one of the most important factors fueling political discontent, frustration and distrust among society (video malaise)
15	Gerbner & Gross, 1976 [31]	The high rates of viewing violence on television, video games, Internet and social networks lead to a higher rate of violent behaviour, and a higher level of anxiety and fear towards the future. Spreading false content through the media causes panic, extremism, and sowing division
16	Jamieson & Romer, 2014 [32]	
17	Ibrahim, 2015 [33]	
18	Higdon, 2020 [34]	
19	Bahaa, 2022 [35]	
20	Noelle-Neumann, 1973 [47]	Most people look at any issue from the media's perspective, and those who disagree with the majority's opinion choose to remain silent.
21	Noelle-Neumann, 1974 [48]	
22	Noelle-Neumann, 1977 [49]	
23	Ball-Rokeach & DeFleur, 1976 [50]	The media as social systems are a constituent part of modern societies and link them to other social economic and political units in a relationship of mutual dependence that results in cooperation and conflict at the same time.
24	DeFleur & Ball-Rokeach, 1989 [51]	
25	Rushti, 1978 [52]	Media functions: Achieving social cohesion, transmitting heritage, leading social change, presenting positive models in public affairs, literature, arts and culture, monitoring the interests and goals of society, and education. Undesirable effects: Increased anxiety and agitation. The role of the units is functional if it helps to stabilize the system and maintain its balance. Dysfunctional occurs if the behavior practiced by the units disturbs the stability of the social system and impedes its progress.
26	Makkawi, & Al-Sayed, 1998 [53]	
27	Abdulrahman, 1984 [54]	Media functions: Alleviating the social conflict within the community, reducing the symptoms of alienation. Underestimating the alternative social and economic systems to the capitalist system. Achieve maximum profits
28	Vallone, Ross & Lepper, 1985 [55]	Hostile Media Effect: The influence of an elite with political orientations critical of the media leads to a lack of confidence in what the media publishes and leads individuals to believe that the media's coverage of controversial issues is hostile and biased against them, even if that coverage is characterized by a great deal of balance and impartiality.
29	Watts, Domke, Shah & Fan, 1999 [56]	
30	Postman, 1985 [57]	Propaganda is “the most mischievous word”. Television destroys the seriousness and rationality of public discourse.
31	Herman & Chomsky, 1988 [58]	Mass media manufacture popular acceptance through its propaganda, with the aim of promoting dominant ideologies and protecting political and economic interests. Propaganda succeeds in transforming public opinion from being pacifist and uninterested in war to being hysterically supportive of war
32	Chomsky, 1997 [59]	
33	Mullen & Klaehn, 2010 [60]	
34	Alford, 2017 [61]	
35	Alford, 2018 [62]	
36	Becker & Roberts, 1992 [63]	Servicing the Economic and the Political System
37	Shamir & Shamir1997 [64]	Media can be a factor of “Pluralistic Ignorance” and misreading of the majority position
38	Nada, 2002 [65]	
39	Nada, 2012 [66]	
40	Alquaary, 2021 [67]	
41	Newton, 1999 [68]	Media has a negative role in political life by widening the circle of discontent, and a positive role in political mobilization.
42	Ejaz, 2017 [69]	
43	Schuck, 2017 [70]	
44	Fleeson, 2000 [71]	Investigative reports focus on social justice, accountability, explaining complex social problems, exposing corruption and abuse of power, serving the public interest, enriching societal debate, and stimulating positive change in society.
45	Forbes, 2005 [72]	
46	Kaplan, 2013 [73]	
47	Potter, 2004 [74]	News Media Literacy is an important requirement to counter the rapid and effective spread of false and misleading information.
48	Spikes & Rapp, 2022 [75]	
49	Okela, 2022 [76]	
50	Thoman, & Jolls, 2005 [77]	Media literacy is the appropriate educational approach for the twenty-first century. It builds a sound understanding of the media's role in society and provides the basic skills of scrutiny and self-expression in the democratic societies.
51	Hall et al., 2005 [78]	The media function: Supporting the dominance of elites in society. More dependency and cultural hegemony in favour of developed countries.
52	Al-Batariq, 2007 [79]	
53	Entman, 2007 [80]	Framing is a form of media bias, in addition to setting the agenda within the role of the media in distributing power. The danger of media framing is increasing in the atmosphere of conflict and polarization, as the media plays dangerous roles in increasing division and mobilizing the conflicting parties towards further aggravation and demonization of the other.
54	Al-Sanjari, & Al-Dawoudi, 2019 [81]	
55	McQuail, 2010 [82]	Media functions: information dissemination, interdependence, social cohesion, continuity in social communication between sub-cultures within society, leisure and entertainment, mobilization in various political, war, economic and religious fields. The media as institutions of society. The media perform the necessary tasks of order, control and cohesion. They are also important for achieving adaptation and change, management of tension. Democratic Participant theory emphasizes the prevention of monopolies, the co-presence of vertical and horizontal communication, and the promotion of equality. The tidal wave of irresponsible information is a threat to social cohesion both within and between countries. This makes media literacy, even at a beginning level, all the more important to generalize in a population.
56	Heyneman, 2021 [5]	
57	Press & Williams, 2010 [83]	The role of Hollywood cinema in the tepidity of the critical consciousness, especially among the working classes, and the acceptance of the humiliating situations in a comic style
58	Hobbs, 2010 [84]	Media and information literacy to achieve the sustainable development and promote democracy, to put a political pressure on governments, companies and non-profit institutions, to speak on behalf of absent voices in the societies, and to confront the extremism.
59	Al-Rawi, 2016 [85]	
60	Kahne & Bowyer, 2019 [86]	
61	Wilson et al., 2011 [87]	Media and Information Literacy to acquire the core competencies of knowledge, skills, and attitudes that allow citizens to interact with media and other sources of information effectively, and to develop critical thinking and lifelong learning skills for the socialization that makes them active citizens.
62	Chen et al., 2011 [88]	The characteristics of new media require that the user become “a critical prosumer”
63	Gregory, 2015 [89]	Citizen Investigators are eyewitnesses documenting human rights abuses, posting through social networks, and being picked up by traditional media. They use investigative techniques to expose malpractices, exposing the hidden in the service of the public interest.
64	the GIJN guide, 2019 [90]	
65	Eşitti, 2016 [91]	With “narcotizing Effect of Social Media” people become like “passive protesters”. “Slacktivism” means activity and idleness at the same time, It is a combination of the words ‘activism’ and ‘slacker’, meaning that spending a lot of time reading and writing posts and tweets may narcotize people rather than motivate them to take real action on the ground.
66	Dubai Media City and Dubai Press Club, 2016 [92]	The impact of a limited number of Social Media Influencers (SMI) may lead to a monopoly of influence.
67	Ezzat, 2020 [93]	
68	Owes, 2017 [94]	The more the individuals follow and trust the mobile media, the less they feel political helplessness, isolation, and apathy.
69	Wardle & Derakhshan, 2017 [6]	Information Disorder = Misinformation, Disinformation, Malinformation
70	Park, 2017 [95]	Citizen podcast producers perform the function of explaining and interpreting social issues and sometimes play the role of critical commentator on the practices of governments and business companies, and in this sense, they play an alternative or complementary role of the professional journalism.
71	Laria & Mar'i, 2017 [96]	Mobile journalism enables professional journalists and citizens to overcome some challenges and promotes the freedom of expression and the right of access to information. It allows the audience to produce high quality media materials.
72	The Code of Practice on disinformation, 2018, 2022 [97]	Initiatives to cut financial incentives for promoters of disinformation. Transparency of political advertising. Empowering users with enhanced tools that help them understand, identify, and report misleading content. Empowering researchers and enhancing the information verification capabilities.
73	Middaugh, 2018 [98]	Civic media literacy means the ability of using media to achieve civic goals and democratic principles, The equal opportunity to interact with the media messages cannot occur if disinformation prevails.
74	Middaugh et al., 2022 [99]	
75	Mutsvairo & Bebawi, 2019 [100]	Teaching “fake news” as an independent course not only for media students, but also for political science and sociology students, because the impact of this phenomenon is not limited to journalists.
76	Potnis, Jasmin, & McLenan, 2019 [101]	Investigative citizens face many difficulties, including accessing to the Internet in some countries, media and information illiteracy, the lack of knowledge of corruption and its risks, corruption illiteracy, the lack of information security, the lack of open sources of information, the lack of confidence in journalists and governments, and the lack of a supportive political, cultural, and legal infrastructure.
77	McDougall, 2019 [102]	Digital and e-Health literacy enables society to efficiently search for information, maintaining a critical vision and an open mind, and using up-to-date knowledge to improve mental health. It measures the level of individuals' knowledge of the useful information sources on the Internet, as well as the usage of that information to combat diseases and epidemics and to confront stress, anxiety and depression.
78	Abdulai et al., 2021 [103]	
79	Bosanac & Luic, 2021 [104]	
80	Patil et al., 2021 [105]	
81	Luo & Harrison, 2019 [106]	Citizen journalism has an impact on traditional media agendas and government policymaking.
82	Seth & Singh, 2019 [107]	Mobile journalism can help promote democracy and express people's ideas in the most efficient way and with the least amount of worries. It can help traditional media achieving their goals. The main element of mobile journalism is the audience, as “hypermobile citizens” can obtain the same benefits that were previously exclusive to journalists.
83	Perreault & Stanfield, 2019 [108]	
84	Bui & Moran, 2020 [109]	
85	King, 2019 [110]	Social media play a role in narcotizing users by providing a negative and effortless alternative. The repercussions of this effect are harmful to the democratic process, especially with the low rates of political participation.
86	Mateus, 2020 [111]	The constant flood of information may arouse a lethargic feeling, a numb, drowsy sensation. The intense media experience has a psychological dimension, although the availability of information is beneficial to the society.
87	Hobbs, 2021 [112]	The dark side of propaganda is the dissemination of lies, hate speech and terrorism. The useful propaganda in the fields of arts, social activities, public service announcements and elections. Education about contemporary propaganda is essential for all ages.
88	UNESCO, 2020 [113]	The world has faced a second pandemic besides Covid-19, which is the “disinfodemic”. It requires combating misinformation, conspiracy theories and discrimination, promoting best communication practices in the face of the crisis, a campaign to support distance education, identifying misleading information and ensuring safety on the Internet.
89	UNICEF, 2020 [114]	
90	Salma, 2020 [115]	News verification by ensuring that the published material is not fabricated or used in the wrong context. Fact-checking focuses on increasing the ability to examine allegations made by officials and decision-makers, raising the level of awareness of community members, increasing their immunity against misleading information and rumors. It has trained them to be more able of holding published allegations accountable.
91	Grizzle et al., 2021 [116]	Media and information literacy is the term recently adopted by UNESCO.
92	Hamouda, 2021 [117]	Factors make it easier to fall victim to disinformation operations: The individuals lock their selves within narrow prejudices, emotional distraction from anxiety or sadness, they are not adopting active open-minded thinking methods.
93	Yinkassar, 2021 [118]	Digital silence is one of the manifestations of imbalance in communication. Fear of isolation and anonymity to comment on the web news are the factors that affect the desire of individuals to publish their own opinions. Although social networks provided greater opportunities for expressing opinions, individuals remain silent when they feel they represent the opinion of the minority.
94	Mahmoud, 2021 [119]	Risk Perception Theory: There is a correlation between the rate and patterns of exposure to negative news and the recipient's sense of collective danger. The media draw the attention to collective threats and dangers, warn of crises or challenges related to national security. Dysfunction occurs when the media overreact based on the conspiracy theories.
95	Hughes et al., 2022 [120]	
96	Xiao & Su, 2022 [121]	Intense exposure to fake news increases misperceptions and leads to more sharing of misinformation. Media literacy skills and critical consumption of communication messages contribute to reducing the odds of falling prey to misinformation or sharing fake news.
97	Mansoor, 2022 [122]	
98	UNESCO, 2022 [123]	Media and information literacy is a sustainable skill development activity in order to enhance trust, social protection and collective solidarity. The establishment of media and information literacy is an essential element for the human rights.
99	Nguyen, 2022 [124]	Developing a model for the patriotic and media education curricula based on Herman and Chomsky's propaganda model (1988) [58].
100	Peng, Lim & Meng, 2022 [125]	Online disinformation uses dozens of persuasive strategies in the field of health.
101	Edelman Trust Barometer, 2022 [2]	“The world ensnared in a vicious cycle of distrust, fueled by a growing lack of faith in media and government. Through disinformation and division, these two institutions are feeding the cycle and exploiting it for commercial and political gain.”

**Table 3:** Open coding.

Functions: (expected roles)	Dysfunction: (adverse effects)	Opportunities
Surveillance of the environment. Interpretation. Heritage transmission. Integration of subcultures into society. Social cohesion. Alleviating social conflict, management of tension. Education, culture, and socialization. Modernization. societal debate. Maintaining social standards. Entertainment. Political advertising and election campaign. Commercial advertising and public service announcements. Persuasion and public relations. Mutual dependence with other social institutions. Serving the existing political and economic system, political mobilization, drawing attention to threats, collective risks, and crises (without exaggeration). Oversight of society's interests. Adaptation, The integration of immigrants and the reduction of symptoms of alienation. Investigative coverage to expose the corruption and serve the public interest. Democratic participation and equality in expressing opinion.	Information disorder, disinformation and fake news. Manufacturing consensus and acceptance. Pseudo - environments support the dominance of the elite. Media and cultural dependence on the strongest. Frustration and discontent. Narcotizing and acceptance of negative situations. Negative protest in networks. Knowledge gap. Detachment. Refrain from political participation. Decreased trust in governments and the media. A threat to social cohesion both within and between countries. Anxiety, fear and isolation, cynical rejection. Hate speech, terrorism, extremism and violent behaviour. Raising panic and spreading conspiracy theories. Sowing division and demonization of the other. Remain silent (digital silence). Making a profit is the most important. The hostile view of the media. Transforming public opinion from pacifist to pro-war. Pluralistic Ignorance, misreading of the majority position. Bias and framing. The monopoly of the influence by a few digital influencers and celebrities.	Media and Information Literacy. News verification and fact-checking. Education is the optimal approach to tackling fake news as a “vaccination”. Combating fake news and disinformation would reduce the temperature of political and social debates and improve trust in societies. Social networks provided more opportunities for expressing opinions. Active and supportive international institutions.
Challenges	Stakeholders (individual/entities)	
Difficult access to the Internet and social media in some countries, Media and information illiteracy. Lack of confidence in the media and governments. Lack of a supportive political infrastructure. Lack of a supportive cultural infrastructure. Lack of a supportive legal infrastructure.	Citizens. Journalists. Governments. Parties. Leaders. Elites and celebrities. Social media influencers. Citizen journalist. Investigative Citizen. Citizen podcasts. Social networks. Media institutions. Economic institutions. Education and culture institutions. other social institutions.	

**Fig. 2:**
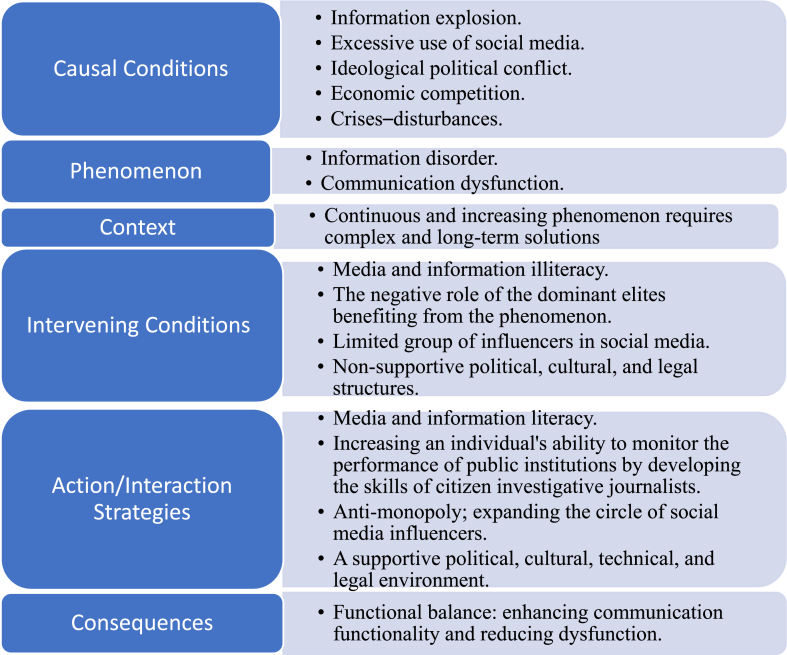
Axial coding.

In the axial coding (Fig. 2), the ideas were regrouped as a phenomenon, consequences, context, intervening conditions, and action/interaction strategies, based on the grounded theory methodology.

In the final stage of coding (selective coding), the theory is integrated and refined. This stage highlights the “central category”, which needs to be a variable that has analytic power in order to “pull the other categories together to form an explanatory whole” [10] (pp.143,146). One category was selected from the categories that emerged during the axial coding to be the central category around which the phenomenon is interpreted to answer the research question.

## 3 Results

To identify the main ideas related to the research topic included in previous literature, at least one main idea from each reference was recorded in chronological order, and then similar ideas were regrouped into boxes regardless of publication date, as shown in Table 2, for example, the first box contains the main ideas from references [1,30]. Another box has related ideas from Refs. [[31], [32], [33], [34], [35]].

### 3.1 Open coding

Based on the initial step of identifying the key ideas presented in the previous literature (Table 2), open coding was conducted (Table 3) by re-reading those ideas across five parts related to the study's topic, which were as follows:

- Media functions or the expected roles;
- Communication dysfunction or the harmful effects;
- Opportunities to enhance the functions and to reduce the dysfunction;
- Challenges;
- Relevant stakeholders.

### 3.2 Axial coding

In this stage, coding was conducted using the paradigm model of axial coding, which consists of six parts (Fig. 2). This stage builds upon the initial open coding stage.

The ideas, elements, and categories that emerged from the open coding were reassembled in this coding stage. For instance, detailed aspects related to the media functions and the communication dysfunction were not explicitly apparent in this coding stage. Instead, they were grouped together as phenomena and consequences within a coding model that also addressed the contextual, causal, and intervening conditions, as well as the action and interaction strategies.

### 3.3 Selective coding

In the final stage of selective coding, a core category was identified within the action/interaction strategies that were elucidated in the axial coding stage. This core category revolves around enhancing media and information literacy (MIL).

The central question in this stage of coding is as follows: How can the enhancement of MIL skills promote communication functions and mitigate dysfunctional aspects? How can MIL address the intervening conditions outlined in the axial analysis framework?

**Fig. 3:**
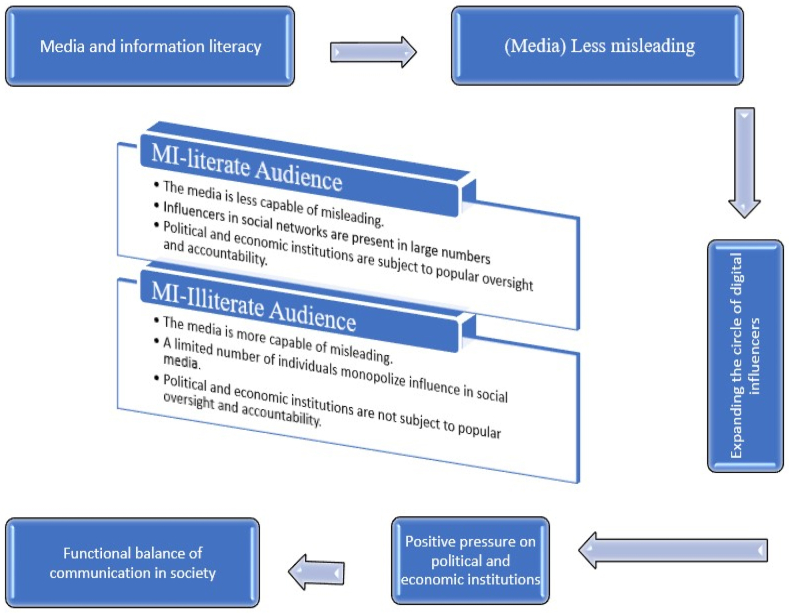
Selective coding: MIL and the functional balance of communication.

Fig. 3 shows that MIL can play a critical role in increasing an individual's ability to resist disinformation and enhance their investigative and supervisory skills over the performance of institutions by developing citizen journalist and investigative skills, as well as in combating monopolies and expanding the circle and number of influencers in social media.

To further clarify the core idea of the final coding stage, Fig. 3 demonstrates how a comparison can be made between an audience possessing high levels of MIL skills and an audience lacking such skills. The comparison explores their relationship with the media, political and economic institutions, and social media influencers.

This model (Fig. 3) is based on the third stage of analysis (selective coding), which focused on one pivotal idea captured during the second stage of analysis (axial coding). The model addresses the phenomenon of dysfunction in communication from both the media and educational perspectives, and it explains how MIL can achieve balance in the relationship among the public, the media, the dominant institutions, and the digital influencers. However, the model does not address the whole intervening conditions that contribute to communication dysfunction, such as the weakness of the supporting political, cultural, and legal structures. These factors and their consequences necessitate more complex and diverse models and recommendations that involve many parties in society and are not limited to educational and media institutions.

## 4 Discussion

This study employed the methodology of a grounded meta-synthesis, which relies on induction to reach a new model in accordance with the study's objective. The coding results, particularly in the early stages, revealed multiple dimensions of communication functions. If “function” represents the positive role that the media outlets are expected to fulfill, then “impact” refers to the actual realized role, whether positive or negative.

### 4.1 Communication functions and dysfunctional aspects

Communication functions are the expected roles that the media should fulfill for the benefit of society. On the other hand, dysfunctional aspects encompass the harmful effects or functional inadequacies resulting from internal structural reasons or external factors related to the relationship with other social units.

Researchers have long been interested in exploring communication functions within society (see Table 2). These functions include the surveillance of the environment; the integration of social units; the intergenerational transmission of social heritage; intercultural communication; socialization; the maintenance and reinforcement of social standards; the modernization of developing societies; entertainment; propaganda in various political, military, economic, and religious fields; and, sometimes, the dominance of influential elites within society, serving the existing political and economic system [4,36,38,63,82] [39,40,43].

Determining the functions of the media and communication in any society is closely linked to the prevailing cultural, political, and social philosophy. This explains, to a large extent, the variation in emphasis among researchers, with some focusing on certain functions while neglecting others, influenced by their philosophical backgrounds and beliefs. For example, writings by critics have emphasized the function of the media in supporting the dominant elites in society [78]. Additionally, scholars influenced by the Frankfurt School and Marxist thought have highlighted the phenomenon of critical consciousness depletion among the working classes. They argued that entertainment and Hollywood films encourage audiences to accept humiliating conditions in a comedic style [83].

As social units, media outlets perform a range of recurring activities that fulfill various functions to serve societal needs. The relationship between the media and other social institutions is based on mutual reliance to ensure societal stability and balance. Dysfunction refers to the media's failure to fulfill its expected roles for the benefit of society, sometimes leading to negative roles. It could be the absence of the hoped-for positive role or the negative roles.

Researchers in this field have written about the narcotizing dysfunction of mass media and social media resulting from the excessive exposure or use of these communication channels. Instead of stimulating and motivating the public to engage positively, they numb them. With increased exposure to the media, people tend to become apathetic and struggle to navigate the overwhelming amount of information they receive. This constant information flood may induce a sense of inertia and narcotization, leading to the possibility of withdrawal and cynical rejection towards the political institutions in society. These negative effects have a detrimental impact on the democratic process, especially with declining rates of political participation [38,45,91,110,111].

Communication dysfunction manifests in various forms and can reach alarming levels of inciting resentment, dissatisfaction, violence, extremism, panic, and fear within society [31,34,46,56]. It may take the shape of bias, framing, or illusion making [64,80,81]. It can also manifest as a reinforcement of silence, whether in the era of traditional media [[47], [48], [49]] or digital silence in social media [118].

Communication dysfunction has been expressed by the term “information disorder”, which is spreading in various forms. Throughout the past century, scholars have addressed communication dysfunction and its multifaceted and complex manifestations. Walter Lippmann indirectly referred to this when he wrote about “the function of news and the function of truth” [1] (p.358). He also indicated the flaws in journalistic performance, stating that it is subject to the advertisers and dominant powers. He argued that leaders, governments, and political parties manipulate the public through the manufacturing of consent and the creation of simplified symbols to interpret the complex world as pure good or absolute evil.

The dissection of media dysfunction is based on the following elements. Some of them were indicated by Walter Lippmann and later elaborated by the Ball-Rokeach and DeFleur model (1976) [50]. These elements can be viewed from new angles that suit the new reality of communication in the internet environment:

1. Traditional and new media;
2. Social institutions, especially the influence of political and economic institutions on media performance;
3. The audience, particularly the prominent role of citizen journalists and digital influencers.
Instances of communication dysfunction are often attributed to the negative or monopolistic roles played by one or more of these three parties. Thus, the following question arises: How can a balanced relationship be established among these three parties, ensuring that none dominates or monopolizes the communication landscape in society?

### 4.2 Media and information literacy (MIL)

The current study, during its open and axial coding stages, explored the diverse and intricate aspects of media dysfunction. Accordingly, solutions should be complex and multifaceted, encompassing the rectification of political, cultural, and legal structures within society.

From communication and education perspectives, the axial and selective coding processes revealed that MIL is the key to enhancing individuals' and communities' capacities to address communication disorders. MIL empowers individuals and communities in three main aspects:

1. Disinformation resistance;
2. The investigative and supervisory skills over the performance of the dominant institutions in society;
3. Combating monopolistic influences in social media.

Although Walter Lippmann did not use the term “media and information literacy”, which has become popular in recent years, he emphasized that education is “the supreme remedy” to address the public's susceptibility to manipulation and the negative role of journalism, political institutions, and economic forces in society. However, he considered education an elusive solution at the time and proposed quick remedies, such as establishing independent bodies of experts to address these imbalances and providing alternatives to decision makers based on information gathering and analyses. Lippmann also highlighted the role of education in teaching students about the habits and skills of information source examination, and questioning whether journalists have actually witnessed the events they report. They can also be taught how to analyze the descriptions of other events; to understand the characteristics of censorship, privacy, propaganda, and the prevailing stereotypes; and to comprehend how symbols impose specific patterns on the imagination. The teacher can also explain how symbolic stories are constructed, how relationships are interpreted, and how abstractions are embodied [1] (pp.408-409).

Before the term “media literacy” emerged, a number of writers and thinkers from different disciplines were interested in how to educate people to protect themselves from the manipulative power of language. One notable figure is Neil Postman (1931–2003), known for his critical writings on the media. Postman focused on language, the media, and cultural teaching methods, arguing that propaganda is “a most mischievous word” and criticizing the television's role in destroying seriousness and rationality in public discourse [57,112].

The definition of media literacy has evolved to correspond to the development of communication and cultural environments. The concept has expanded to include not only immunization against harmful communication messages, but also the production of critical messages.

Media literacy is a 21st century approach to education. It provides a framework to access, analyze, evaluate, and create messages in a variety of forms, from print to video to the Internet. Media literacy builds an understanding of the role of media in society as well as the essential skills of inquiry and self-expression necessary for citizens of a democracy [77] (p.21).

For example, news media literacy is an essential requirement in combating the rapid and influential spread of false news and misinformation. It provides individuals with skills and competencies that enhance their need for knowledge and enable them to recognize media (news) content, understand the structure of the media industry, perceive media influence, and comprehend their own identity and the surrounding environment [[74], [75], [76]]. Civic media literacy supports the ability to use media to achieve civic goals and democratic principles. Equal opportunities to engage with communication messages cannot exist if the deception prevails [98,99].

Empowering citizens with MIL enhances the effectiveness of their political participation in positively lobbying governments, businesses, and non-profit organizations. When they become aware of personal, corporate, and political agendas, they can speak out on behalf of the missing voices and omitted perspectives in our communities. By embracing the values of media literacy, social balance and stability among different religions and sects can be fostered. By focusing on elements and values that unite people rather than divide them, it is possible to confront online religious extremism [[84], [85], [86],^116^]. The meanings and aforementioned goals affirm that MIL is not only necessary for achieving balance in the relationship between the audience and the media, but it has also become more closely associated than ever before with the requirements of comprehensive development and the indicators of quality of life sought by modern societies.

Heyneman (2021) proposed that public schools throughout the world consider a certificate of media competence, might be used as a criterion for instance, in an application for employment in the civil service or enter the military [5]. Dealing with fake news and disinformation would dial down the temperature of political and social debates – and confrontations – would improve trust in societies [30].

The phenomenon of “citizen journalism” as one of the manifestations of the modern technological leap in the world of communications and information has enabled the public to assume the role of the fifth estate, which monitors the performance of the four powers in society: executive, judicial, and legislative powers, and the traditional media. Citizen investigators include “ordinary citizens, members of nongovernmental organizations, and non-journalism professionals interested in using investigative techniques to uncover wrongdoing and expose the invisible” [90] (p.2).

These advanced skills of citizen investigators empower them to monitor dominant institutions and figures in society and exert positive pressure on them. This cannot be achieved without advanced MIL skills, alongside political and legal education and the technological skills associated with utilizing available sources of information on the Internet. Consequently, citizen investigators who possess a high degree of MIL are expected to actively contribute to preventing political and economic institutions from monopolizing the communicative influence in society and achieve a certain level of functional balance.

The phenomenon of social media influencers is also related to this. Their influence has become tangible not only in the field of commercial campaigns and advertising, but also in social and cultural issues. Concerns are growing that a limited number of them are monopolizing the influence, especially on teenagers. Digital influencers play an indirect role in influencing their fans, shaping their digital identities, motivating them to change their attitudes and behaviors towards events, and adopting certain lifestyles [93]. Therefore, working to increase the number of social media influencers equipped with MIL skills would contribute to reducing influence monopolization and achieving diversity. In fact, this goal is strongly consistent with the democratic participant theory, which stresses the importance of preventing monopolies, providing the co-presence of vertical and horizontal communication, and promoting equality [82] (pp.151-152).

## 5 Conclusions

In conclusion, achieving balance in the relationship between different social units is a complex issue that is influenced by internal and external cultural, religious, social, political, and technical factors. Providing a standardized model or a simplified solution is challenging. This study sought to present a model for only the communication aspect within that relationship, which included four main parties: the audience, the media, the political and economic institutions, and the digital influencers.

The proposed model was not built from scratch, but rather relied on a rich heritage of over a hundred years of scientific and professional accumulation by communication researchers and practitioners. The study employed open, axial, and selective coding to explore the functions of communication, the aspects of dysfunction, the opportunities, the challenges, the action strategies, the interactions, and the relevant entities. It ultimately identified MIL as the core category in the final stage of the coding process.

The new model proposed by this study on MIL and its role in the functional balance of the media and communication performance in society was based on grounded meta-synthesis methodology. This methodology has clear steps for data collection and coding, aiding in building new models and theories. Its philosophical framework (critical realism) not only theorizes and represents information and relationships, but also provides decision makers in relevant bodies with important signals for the possibility of action and changing reality for the better. It is important to note that this model can only succeed in a supportive and protective political, cultural, technological, and legal environment.

Furthermore, the new model opens the door to new research questions and hypotheses that can be tested in future studies. For example, what are the intervening variables that may impact the relationships within the MIL model proposed by this study? Will expanding the circle of digital influencers help reduce the information disorder phenomenon? How can ordinary citizens benefit from the principles of MIL and mobile journalism skills and become investigative citizens? What are the ethical considerations that should be emphasized in this context?

The model presented in this study should be interpreted within the framework of the methodological and data limits. By reviewing 101 studies, books, and reports published over the last century, the study attempted to extrapolate a complex and extended communication phenomenon. This sample, However, may not be large enough to make a final and comprehensive evaluation. Furthermore, the proposed model focuses on the media and educational dimension, while additional research into the phenomenon of communication dysfunction and information disorder is required from the cultural, political and legal perspectives.

## Data availability statement

This is a meta-synthesis review, so the data were detailed and referenced in the manuscript.

## CRediT authorship contribution statement

**Hasan M.H. Mansoor:** Writing – review & editing, Writing – original draft, Methodology, Investigation, Funding acquisition, Formal analysis, Data curation, Conceptualization.

## Declaration of competing interest

The author declared no potential conflicts of interest with respect to this article.
